# Qualitative Analysis of E-Liquid Emissions as a Function of Flavor Additives Using Two Aerosol Capture Methods

**DOI:** 10.3390/ijerph15020323

**Published:** 2018-02-13

**Authors:** Nathan Eddingsaas, Todd Pagano, Cody Cummings, Irfan Rahman, Risa Robinson, Edward Hensel

**Affiliations:** 1 College of Science, Rochester Institute of Technology, Rochester, NY 14623, USA; ncesch@rit.edu; 2 National Technical Institute for the Deaf, Rochester Institute of Technology, Rochester, NY 14623, USA; tepnts@rit.edu (T.P.); cxc6820@rit.edu (C.C.); 3 UR Medical Center, University of Rochester, Rochester, NY 14627, USA; Irfan_Rahman@URMC.Rochester.edu; 4 Kate Gleason College of Engineering, Rochester Institute of Technology, Rochester, NY 14623, USA; rjreme@rit.edu

**Keywords:** electronic cigarettes, emissions, regulatory science, flavor, harmful and potentially harmful constituents, e-liquid, tobacco product characteristics

## Abstract

This work investigates emissions sampling methods employed for qualitative identification of compounds in e-liquids and their resultant aerosols to assess what capture methods may be sufficient to identify harmful and potentially harmful constituents present. Three popular e-liquid flavors (cinnamon, mango, vanilla) were analyzed using qualitative gas chromatography-mass spectrometry (GC-MS) in the un-puffed state. Each liquid was also machine-puffed under realistic-use flow rate conditions and emissions were captured using two techniques: filter pads and methanol impingers. GC-MS analysis was conducted on the emissions captured using both techniques from all three e-liquids. The e-liquid GC-MS analysis resulted in positive identification of 13 compounds from the cinnamon flavor e-liquid, 31 from mango, and 19 from vanilla, including a number of compounds observed in all e-liquid experiments. Nineteen compounds were observed in emissions which were not present in the un-puffed e-liquid. Qualitative GC-MS analysis of the emissions samples identify compounds observed in all three samples: e-liquid, impinge, and filter pads, and each subset thereof. A limited number of compounds were observed in emissions captured with impingers, but were not observed in emissions captured using filter pads; a larger number of compounds were observed on emissions collected from the filter pads, but not those captured with impingers. It is demonstrated that sampling methods have different sampling efficiencies and some compounds might be missed using only one method. It is recommended to investigate filter pads, impingers, thermal desorption tubes, and solvent extraction resins to establish robust sampling methods for emissions testing of e-cigarette emissions.

## 1. Introduction

This work is built upon the premise that current protocols commonly employed for testing alternative tobacco products, such as e-liquids used as the consumable product in conjunction with electronic cigarettes (e-cigs) may be insufficient to qualitatively identify all harmful and potentially harmful constituents (HPHCs) present in the aerosols generated by vaporizing such e-liquids. Emissions test standards must be developed for the identification of HPHCs. We further assert that such protocols must involve testing both the un-puffed consumable (i.e., e-liquid) and the aerosols generated under realistic (representative of natural-use environment topography) puffing conditions. While robust protocols have been established for testing conventional cigarettes, uniform standards for testing e-cigs and water pipes have not been adopted, though several variations have been proposed [1]. While there is a fair amount of literature available on e-cig emissions, the variations in product characteristics and testing conditions make it difficult to leverage published data to inform a comprehensive understanding of the emissions as they relate to the characteristics of the un-puffed e-liquid. The United States’ Food and Drug Administration (FDA) recommends both non-intense and intense protocols for evaluating e-cig emissions, and testing e-liquids with low and high emission devices, but actual protocols are not specified. There is currently little consistency in puffing regimes being used for Electronic Nicotine Delivery System (ENDS) emission studies, including 15 mL/s, 4 s puffs [1], 27 mL/s, 3 s puffs [2], 39 mL/s, 1.8 s puffs [3], 27.5 mL/s, 2 s puffs [4,5], 17.5 mL/s 2 s puffs [6,7,8], 10 mL/s, 4 s puffs [9], and in some articles the puffing protocol is unclear [10,11].

Sampling techniques also vary among previously reported studies; including Cambridge filter pads [1,4,6], EpiAirway™ tissue cultures [2], Teflon filters [10], glass fiber filters [7], quartz fiber filters [12], impingers [5,11], polyurethane foam [8], solvent extraction resins [3], and thermal desorption resins [9,13]. Additionally, several commercially available puffing machines, including Vitrocell [2,4,6], Cerulean [5], Borgwaldt [7], Teague Enterprises [10], and CH Technologies [8], and some non-commercial machines [1,3,9] have been employed to generate emissions. A number of prior reports have also qualitatively determined the composition of e-cig emissions using a single type of sampling technique [9,13,14,15]. The majority of prior studies do not report attempts to validate the puffing machine itself, and its interaction with the e-cig being tests. Scientific data to support emissions sampling protocols for ENDS are lacking, leaving current regulations open to interpretation. Regulatory guidance informed by actual-use device-specific puffing regimes, scientific studies on appropriate emissions collection methods, and rigorous protocols for puffing machine validation are needed.

This investigation compares two methods for sampling the aerosol emissions from electronic cigarettes (e-cigs) for subsequent analysis using gas chromatography-mass spectrometry (GC-MS). A qualitative GC-MS analysis was performed on e-liquid samples before they were puffed, as well as corresponding emissions that were captured during the puffing process using two methods: Cambridge filter pads and a series of two impingers. The study seeks to enhance the understanding of the relationship between emissions capture methods and the compounds qualitatively identified in the emissions relative to the compounds identified in the un-puffed liquid.

## 2. Materials and Methods

Three flavored e-liquid samples were used in this study, including Cinnamon, vanilla, and mango. Each flavor of e-liquid was analyzed (in the un-puffed form) using samples directly from the manufacturer’s containers. Each sample was diluted 1:40 (*v*/*v*) in methanol (HPLC grade) and placed into 1 mL GC injection vials. These diluted samples were injected directly into the GC-MS (Shimadzu QP2020, Shimadzu Scientific Instruments, Columbia, MD, USA).

Each e-liquid was individually puffed through the same reusable e-cig device; composed of an itaste^TM^ MVP 2.0 (Innokin® Technology, Shenzhen, China) with the iClear^TM^ X.I tank (Innokin® Technology, Shenzhen, China), set at 7 watts. The iClear^TM^ X.I tank is a “bottom” coil-style tank, which designed to deliver e-liquid to a heating coil by through fibrous wicks. The first generation RIT PES^TM^-1 programmable emissions system (Respiratory Technologies Lab, Rochester Institute of Technology, Rochester, NY, USA) was programed to control each discrete puff with a duration of 3.5 s and a puff flow rate of 33.8 mL/s, representative of e-cig puffing behaviors measured in the natural environment. While prior studies of natural environment topography monitoring have demonstrated significant variation in puff duration and flow rate during the course of one- and two-week observation periods [16,17,18,19], we eliminated variation in puff flow conditions as a variable in order to focus on the impact of sample capture methods. To prevent contamination of the e-liquid in the coil/wick, a new coil/wick was used for each flavored e-liquid and the tank body was cleaned and dried after each puffing session. Emissions were captured using two methods. Each capture method was tested in duplicate or triplicate, and not consecutively (i.e., not all filter capture trials were done prior to an impinger trial). Identical quantitative GC-MS analysis was conducted on the samples captured via both methods. We observed chromatographic results to be consistent across trials, and reported only those GC-MS peaks which were consistently observed across multiple trials with each capture method. The primary variable controlled in this study was the method of capturing the sample. The secondary variable controlled was the e-liquid, to show that the significance of sample collection method was not unique to the product. The same e-cig unit was used for all trials on both collection methods for each e-liquid, to remove device variability as a factor in the results reported. 

The “pad sample collection method” employed 48 mm silica Cambridge filter pads (Performance Systematix Inc., Grand Rapids, MI, USA) to capture emissions from the air flow path between the exit of e-cig and the PES^TM^-1 system inlet port. Ten pads for each e-liquid sample were exposed to 100 puffs total (10 puffs per filter pad to avoid overloading pads) using the above puffing regime. Filter pads were stored in separate sealed glass jars prior to GC-MS analysis. All ten pads associated with each e-liquid flavor were combined in a in a container with 50 mL methanol. Each sample container was placed on an orbital shaker (200 rpm) for 24 h to break down the filter pad material, followed by a wrist shaker on high speed for 15 min and another 24 h orbital shaking to ensure breakdown. The resultant samples were filtered through a 0.45 µm regenerated cellulose syringe filter and concentrated using solvent blow-off (nitrogen gas) in a 4 mL graduated concentrator to a final volume of 1 mL or less and placed into GC vials. These samples were then directly injected in the GC-MS. The combining of filters and the concentrating the final solution to 1 mL was done so that compounds in trace amounts or with weak signals could be observed during analysis.

The “impinger sample collection method” used two impingers in series to maximize the collection of emissions. Impingers were created from 250 mL graduated cylinders and extra coarse fretted disks which connected between the e-cig exit and the input to puffing machine. Each impinger was filled with 80 mL methanol and the impinger system was cooled in a bath of acetone in dry ice. Samples collected from the loaded impingers were concentrated using solvent blow-off (nitrogen gas) in a 4 mL graduated concentrator to a final volume of 1 mL and placed into GC vials, which were then directly injected into the GC-MS. A Shimadzu GC-MS-QP2020 (Shimadzu Scientific Instruments, Columbia, MD, USA) was used and the method was optimized for our study from that of Hutzler et al. [20], as shown in Table 1.

Chromatogram peaks were qualitatively identified using the 2014 NIST database and only peaks with signal-to-noise ratios greater than 3 were analyzed. We used the mass spectra from the cinnamon, vanilla, and mango samples (e-liquid, impinger, and pad collection) and the 2014 NIST database to identify several molecules of interest that correlate with the carrier liquid and flavoring agents in E-cigarettes, as well as other molecules associated with the smoking of the e-liquid and potential contaminants. Only chromatographic peaks that could be positively identified and associated with e-liquid or emissions from e-cigarette smoking were included in this study. Positive identification was concluded if the mass spectra matched at a level of at least 85% positive match with the mass spectral library and having an appropriate relative retention, as determined by the retention index of each molecule. Most compounds had better than a 95% library match. A number of peaks were positively identified, but not included because they were determined not to come from the e-liquid or the generated aerosol. The source of other compounds was the GC inlet and column and from the sampling lines. Blank runs were used to determine peaks associated with the GC column and inlet. In addition, the blank runs displayed some peaks from the sampling setup that were not associated with the generated aerosol. Compounds that came from the sampling lines were determined if the peak intensity was identical or greater in the second impinger in series as that in the first impinger.

It is worth noting that we also tried a setup in the puffing machine where the aerosol first traveled through a pad and then into the impinger. Using this set-up, the pad samples showed similar molecules to prior pad samples, but the impinger did not show many molecules of interest. It was determined that the serial setup (pads flowing into impingers) was not a viable system.

## 3. Results

### 3.1. Vanilla-Flavored E-Liquid

**Figure 1:**
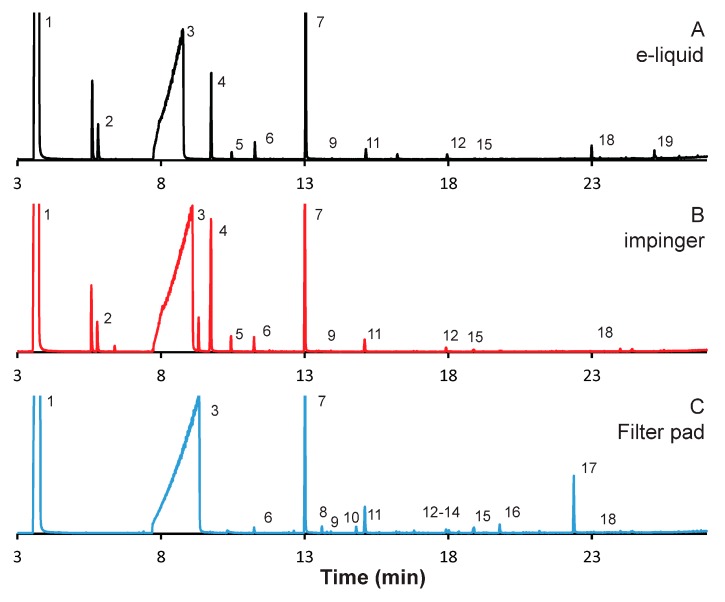
Gas chromatogram (GC) analysis of aerosol emissions resulting from vanilla flavored e-liquid using alternative captured methods. Emissions were collected from 100 puffs under realistic topography conditions. Chromatograms reflect (**A**) e-liquid diluted in methanol, and emissions collected: (**B**) with series impinger 1; and (**C**) on 10 Cambridge pads, with 10 puffs per pad.

Figure 1 illustrates chromatograms of aerosol emissions resulting from the study of vanilla flavored e-liquid. Panel (A) presents the chromatogram of the vanilla e-liquid itself diluted in methanol. Panel (B) presents the chromatogram for the aerosols resulting from the smoking of the vanilla flavored e-liquid, captured with the first of a series of two impingers, while panel (C) presents the chromatogram for the aerosols collected using standard Cambridge filter pads. The identification, retention index, and mass spectral library match of each identified peak, as well as when it was observed and the retention times, can be seen in Table 2. The retention indices are those provided by the NIST 2014 mass spectral database included with the Shimadzu software and is for a DB-1 column, not a DB-17 column as used in this study and, therefore, should be taken as a rough estimate. The more polar the compound, the more the retention index is shifted earlier on a DB-17 column as opposed to a DB-1 column, so compounds such as alcohols will be have less relative retention index values as compared to other less polar compounds. The analysis of interest is to determine what compounds are observed from which sampling technique; to facilitate this, the intensity of nicotine from each chromatogram was normalized to one so comparison of each chromatogram was simpler. Nicotine was chosen because it is observed in all chromatograms and is one of the three main peaks along with propylene glycol and glycerol, the two main components of all of the e-liquids studied. The absolute intensity of the nicotine GC peak from the filter pad sampling run was 15% greater than that from the impinge. The numbers on the chromatograms refer to the compound identification number listed in Table 1. As can be seen, there are a number of peaks not labeled, including a number of prominent peaks. Peaks not numbered have been excluded from the analysis due to poor mass spectral identification, less than 85% match to the mass spectral database, or due to the fact that the compound was known to not be from the emissions from the e-aerosol or e-liquid. The study was not designed to be a comprehensive analysis and there are many other emissions that are produced that were not included due to our criteria, as well as many others that are known to be in very low concentration and/or need different sampling and analysis techniques to study them such as polycyclic aromatic hydrocarbons (PAH). With these stipulations, nineteen compounds were included in the analysis.

Many of the molecules are observed in the e-liquid and each sampling as can be seen in Figure 1 and Table 2. In all, nine of the eighteen compounds included in the analysis were observed in all three cases. There were three compounds that were observed in the e-liquid and the impinger, but not when filter pads were used for sampling. Included in the compounds observed in all three samples were the main components of the e-liquid; propylene glycol and glycerol, nicotine, and the main flavor compound for vanilla; vanillin. As can be seen in Figure 1, the peak for glycerol is very large; this is because it is in such high concentration compared to the other compounds, as it is the main component of the e-liquid. The samples were concentrated in a way so that a number of trace compounds could be observed, resulting in an excess of glycerol. Analysis was performed to determine if the peaks were being obscured by the glycerol peak and no major signals were found. If one were attempting to detect as many species as possible, using GC techniques to reduce the intensity of the glycerol peak could be used, such as using the heart-cutting system with Deans Switch, as has been done previously for the qualitative analysis of e-cigarette emissions using thermal desorption gas chromatography [14]. In addition, there were five compounds that were only observed in the filter pads and one only observed in the e-liquid. There is no chemical commonality between the compounds observed only in the aerosols sampled by the filter pads.

Two peaks were identified as the same compounds, peaks **4** and **5** are both identified as glyceryl 1-monoacetate. There are a number of isomers with very similar structure that all provided a high degree of match within in the mass spectral database and likely one or both or one of the structurally-similar isomers. In addition, a peak at 18.4 min was identified as a long chain aldehyde and not definitively identified. There were a number of possible aldehydes the provided high degree of spectral match so the exact identity was not determined, however, all of the high library matches were linear aldehydes. Standards could be run to determine the identity of the aldehyde.

While the origin of many of the compounds was straight forward, the origin of all of the sampled compounds was not exhaustively investigated. The goal of this analysis was not to identify the source of a given compound but rather to demonstrate that different sampling techniques preferentially sample different compounds. Diethyl phthalate is a known plasticizer and is probably a contaminant from the supplier; however, we cannot rule out sample processing contamination, which we feel is remote, as it is observed in all samples that were processed by different means. Regardless, as it is observed in all samples it is not of great interest to this study as we were interested in the differences between the sampling techniques.

### 3.2. Mango-Flavored E-Liquid

**Figure 2:**
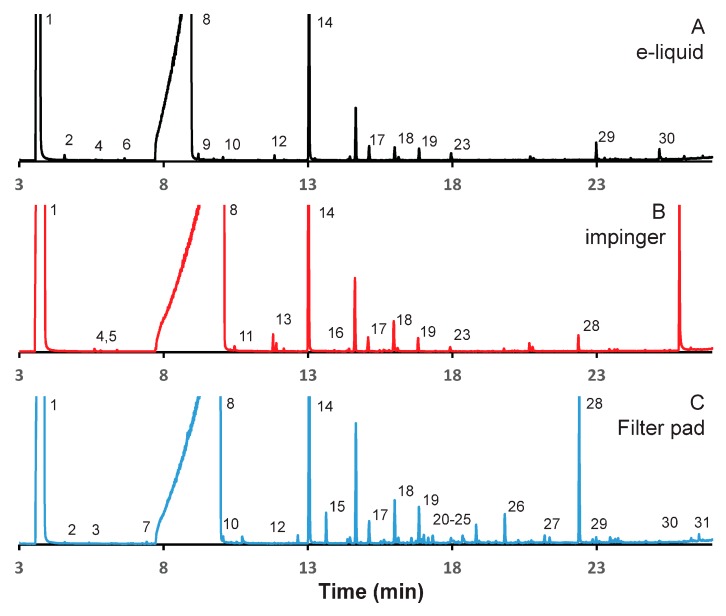
Gas chromatogram (GC) analysis of aerosol emissions resulting from mango flavored e-liquid using alternative captured methods. Emissions were collected from 100 puffs under realistic topography conditions. Chromatograms reflect (**A**) e-liquid diluted in methanol, and emissions collected: (**B**) with series impinger 1; and (**C**) on 10 Cambridge pads, with 10 puffs per pad.

Figure 2 illustrates chromatograms of aerosol emissions resulting from the study of mango flavored e-liquid. As was the case for the vanilla e-liquid trial, all chromatograms were normalized to the peak signal intensity for nicotine for ease of analysis. The absolute intensity of the nicotine GC peak from the filter pad sampling and impinge sampling were nearly identical with less than a 1% different in intensity. Table 3 presents the compounds identified in the mango flavored un-puffed e-liquid, as well as the emissions captured using filter pads and impingers, in all 30 compounds were included in the analysis.

There were more compounds observed and identified from the mango e-liquid and the generated aerosols than from the vanilla e-liquid trials. Similar to what was observed with the vanilla trials, many of the compounds were observed in the e-liquids and from both sampling techniques. In all, seven compounds were observed in all three runs. Five different compounds were observed in the mango e-liquid and in the aerosols sampled by the filter pads and one compounds were observed in the e-liquid and in the aerosols sampled by the impinger, but not the filter pads. One compound was observed in the aerosols sampled by the impinger and the filter pads, four were observed only from the aerosols sampled by the impinger, eleven compounds were only observed in aerosols sampled by the filter pads, and two compound was observed only in the e-liquid. The identification of these compounds can be found in Table 2.

The chemical makeup of the flavor of mango is more complex than that of vanilla or cinnamon and consists of a combination of many compounds. Of the compounds found in mango flavor many were observed here including: hexyl hexanoate, methyl hexanoate, hexyl acetate, citonellol, γ-decalactone, γ-undecalactone, δ-decalactone, and potentially others. Of these, hexyl acetate, γ-decalactone, γ-undecalactone, and δ-decalactone were found in all three samples, citronellol and hexyl hexanoate was observed in the e-liquid and from the filter pad samples while methyl hexanoate was only observed from the filter pad sampling. Due to the fact that all but the methyl hexanoate were observed in the e-liquid it is assumed that methyl hexanoate was not in fact one of the flavors added. It should be highlighted that the impinge samples missed some of the flavor compounds that were included in the aerosol as indicated by the filter pad samples.

Two sets of peaks were identified as the same compound in the mango experiments. Peaks **4** and **5** are both labeled 1,2-propanediol, 2-acetate and peaks **11** and **13** are both labeled glyceryl 1-monoacetate. Similarly to the vanilla trial, a number of isomers with similar structure all provided high degree of match in the mass spectral database, and one or more of the peaks are actually isomers of the identified compound. Peaks **19**, **20**, and **21** (τ-Cadinol, α-Cadinol, and 1-Naphthalenol) were all assigned the same retention index by the mass spectral software. The retention indices are based off using a DB-1 column whereas we used a DB-17 column in this work so the retention index values will not be exact and can vary compound to compound. The fact that all three compounds elute within 15 s of each other provides an additional support to their identity.

As with the vanilla flavor e-liquid samples, a comprehensive analysis of the origin of all emissions was not done as the source of each compound was not the focus of this analysis. Again, the plasticizer diethyl phthalate was observed in all samples and, therefore, of low interest to this study. There were a total of ten compounds only observed in the filter samples including a number of large alcohols, τ-cadinol, α-cadinol, and 1-naphthol, as well as other large compounds, including octadecanal and 9-octadecanamide that we cannot identify the source of. While we cannot rule out contamination as the source, the fact that they are observed reproducibly in subsequent runs and that they are not observed from the other e-liquids indicates that they are associated with this sample.

### 3.3. Cinnamon-Flavored E-Liquid

**Figure 3:**
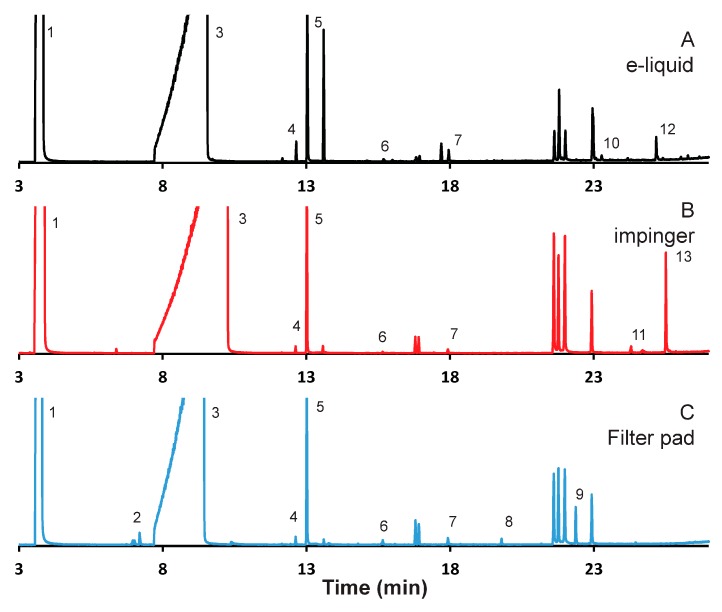
Gas chromatogram (GC) analysis of aerosol emissions resulting from cinnamon flavored e-liquid using alternative captured methods. Emissions were collected from 100 puffs under realistic topography conditions. Chromatograms reflect (**A**) E-liquid diluted in methanol, and emissions collected: (**B**) with series impinger 1; and (**C**) on 10 Cambridge pads, with 10 puffs per pad.

Figure 3 illustrates chromatograms of aerosol emissions resulting from the study of cinnamon flavored e-liquid. As with the other two sets of data, the chromatographic intensities are normalized to that of nicotine. The absolute intensity of the nicotine peak from the aerosols sampled by the impinger was 30% greater than that from the aerosols sampled by the filter pads. As can be seen, a number of prominent peaks in the chromatogram could not be positively identified including a set of peaks observed in all three samples that elute at around 20 min. Table 4 presents the compounds identified in the cinnamon flavored un-puffed e-liquid, as well as the emissions captured using filter pads and impingers. In all, thirteen compounds were included in the analysis.

Many of the compounds identified were observed in all three samples. In total, six out of thirteen identified compounds were observed from the e-liquid, as well as both aerosol sampling techniques including the main components of the e-liquid propylene glycol and glycerol, nicotine, and the main flavor compound for cinnamon, cinnamaldehyde, as well as a small peak from ethyl vanillin, a compound commonly used to give a vanilla flavor. There were two compounds observed only from the aerosols sampled by the impinge, three compounds from the aerosols sampled by the filter pads, and two compounds observed only from the e-liquid.

As with the other e-liquids, there are a number of compounds that were observe that we cannot determine their source. As was observed with the other two e-liquids, the plasticizer diethyl phthalate was observed in all three samples for the cinnamon e-liquid. In addition, another plasticizer, tributyl acetylcitrate, was observed only in the impinger samples. It was observed reproducibly, but we do not know the source and cannot rule out contamination. In addition, tributyl prop-1-ene-1,2,3-tricarboxylate was observed from the impinger samples, but the source cannot be determined at this time.

## 4. Discussion

Thirty-three compounds were positively identified across the range of flavors and sampling methods studied. At least four compounds (Table 5) were positively identified across all flavors and were present in the e-liquid, filter pad, and impinger samples, including propylene glycol, glycerol, nicotine, and diethyl phthalate. Five compounds unique to the vanilla flavor were identified in all three samples, three compounds associated with mango flavor, and one compound unique to cinnamon flavor. Two compounds were observed in the e-liquid and impinger samples (Table 6), but were not evident in the filter pad samples. Three additional compounds were observed in both the e-liquid and filter pads (Table 7), but not in the impinger samples. Three compounds were evident only in the impinger samples (Table 8), while 15 compounds were uniquely identified in the filter pad samples (Table 9). Combined, Table 8 and Table 9 articulate 18 compounds present in the emissions, which were not evident in the un-puffed e-liquid.

Devices such as the one chosen for this study have been reported as prone to overheating the e-liquid on the coil, causing thermal decomposition and various small aldehydes, among other decomposition products that are not originally in the e-liquid. We do not believe the elements were overheating during the experiments conducted here for several reasons, including that each flavor e-liquid had its own heating element, each unit was cleaned thoroughly between trials, each tank/heating element was brand new when used, the heating elements were operated at low power (7.5 W, about the middle range of the unit), and each condition was run in duplicate or triplicate trials, and we reported outcomes only when the same results were observed (the same compounds in the same ratios) each time. We believe each trial was conducted well within the normal operating range of the device, and avoided operating the device at extremes of puff flow rate, duration, volume, and operating power in order to focus attention on the sample capture method. In addition, the compounds that would be observed due to the thermal decomposition of the e-liquid (small aldehydes) include formaldehyde, acetaldehyde, acrolein, benzaldehyde, were not sampled for or detected, and would likely be found in the gas phase, not the particulate phase.

To our knowledge, no other publication has presented similar data documenting the impact of capture system on the accuracy of HPHC identification of ENDS aerosols. Our results demonstrate the sampling method to be an important factor in proper identification of all HPHCs present in emissions from such products. Robust methods for emissions sampling are needed by the tobacco regulatory science (TRS) community, to positively identify HPHCs for toxicology studies, and inform regulation of tobacco product characteristics.

Further investigation is needed to determine optimal aerosol emissions sampling methods to ensure a comprehensive identification of the molecules present in emissions from alternative tobacco product consumables (e.g., e-liquid base, flavor) as a function of user behavior and device product characteristics (e.g., materials, power level, flow path geometry). Each sampling method had advantages and disadvantages related to logistics, including sample preparation and total processing time. Certain collection methods are well suited for high flow rates, while others require more moderate rates; sampling methods have varying capture efficiencies which depend on the class of molecule(s). This study provides the foundation for future analysis, to determine which categories of molecules in aerosol emissions may be prone to capture by either the pad, impinge, or other method as a function of molecular weight, vapor pressure, or other factors.

The qualitative results herein reporting presence, but not amounts, of various components of e-liquids or their aerosols gives little insight into the reasons for differing results arising from variation in the method of sample capture. However, demonstrating that the presence of compounds identified is dependent upon the sample capture method strongly supports our premise that the sample capture method is an important aspect of rigorous e-cig emissions experimental design.

When using the filter pad capture method, each GC-MS qualitative analysis trial was conducted on ten pads, with each pad exposed to ten puffs. While this provided an opportunity for repeated trials across ten pads, we chose to combine all ten pads in order to concentrate the emissions products and obtain a better signal and qualitatively observe more species. The primary outcome of interest to the current study is whether the species identified with pad capture are the same as the species identified with impinger capture, resulting in a binary decision whether each compound is present or not (1 or 0). In this type of experiment design, repeated trials enhance the statistical validity of the binary comparison.

Additional work is needed in the research community to establish robust emissions testing protocols which are accepted as valid by all interested parties. This study focuses on the methods employed to capture emissions samples, which is the first step in every emissions testing and sample analysis protocol. Additional elements of a robust emissions testing protocol require not only a robust sample capture method, but also require study of emissions across a range of flow conditions, devices settings, and operating conditions (such as power) which represent both the intended use of the device, as well as unintended, but observed misuse of the device.

## 5. Conclusions

The results presented here conclusively demonstrate that molecules observed in qualitative GC-MS analysis of e-cig aerosol emissions are dependent upon the capture method employed, particularly for those molecules which are generated in the heating and aerosolization. Qualitative GC-MS analysis of the emissions samples identify compounds observed in all three samples: e-liquid, impinge, and filter pads and each subset thereof. A limited number of compounds were observed in emissions captured with impingers but were not observed in emissions captured using filter pads; a larger number of compounds were observed on emissions collected from the filter pads, but not those captured with impingers. Nineteen compounds were positively identified in the e-cig emissions (three using impinger samples and 16 using filter pad samples) which were not present in the un-puffed e-liquid. It is demonstrated that sampling methods have different sampling efficiencies and some compounds might be missed using only one method. It is recommended to investigate filter pads, impingers, thermal desorption tubes, and solvent extraction resins to establish robust sampling methods for emissions testing of e-cigarette emissions.

## Figures and Tables

**Table 1:** GC-MS operating parameters used for the qualitative emissions analysis results presented herein.

GC-MS Setting	Optimized Method
Capillary Column (GC)	DB-17MS ((50%-Phenyl)-methylpolysiloxane)
Helium Flowrate (GC)	1.00 mL/min
Oven Program (GC)	Ramp 1: 40 °C to 170 °C @ 10 °C/min; hold 2 min; Ramp 2: 8 °C/min to 250 °C; Ramp 3: 25 °C/min to 320 °C; hold 5 min.
Split Ratio Mode (GC)	25:1
Solvent Delay (MS)	0 to 1.00 min
Total Ion Scan (MS)	m/z = (30–450) from 1 min to 32.80 min

**Table 2:** GC-MS analysis of molecules in observed in aerosols generated from 100 puffs of vanilla flavor e-liquid, in emissions captured with pads, and methanol impingers.

Peak Number	Compound	Retention Index	Retention Time	Library Match	E-Liquid	Impinger	Filter Pad
**1**	Propylene glycol	724	3.8	97	X	X	X
**2**	1,2-Propanediol, 2-acetate	864	5.8	97	X	X	
**3**	Glycerol	967	8.5	98	X	X	X
4 *	Glyceryl 1-monoacetate *	1091	9.74	96	X	X	
5 *	Glyceryl 1-monoacetate *	1091	10.45	93	X	X	
**6**	Ethyl maltol	1163	11.27	97	X	X	X
**7**	Nicotine	1341	13.03	97	X	X	X
**8**	Dodecanol	1457	13.63	95			X
**9**	Piperonal	1326	13.95	97	X	X	X
**10**	Decyl acetate	1381	14.83	96			X
**11**	Vanillin	1392	15.13	96	X	X	X
**12**	Diethyl phthalate	1639	17.97	96	X	X	X
**13**	Lauryl acetate	1580	18.05	92			X
**14**	Piperonal propylene glycol acetal	1617	18.92	95	X	X	X
**15**	Methyl hexadecanoate	1878	19.82	95			X
**16**	Methyl stearate	2077	22.4	96			X
**17**	Butyl hexadecanoate	2177	22.99	96	X	X	X
**18**	Butyl octadecanoate		25.17	93	X		

* Note: Compounds **4** and **5** are identified as the same compound but there are many isomers that are very similar that show strong mass spectra correlation. Please see text for further information.

**Table 3:** GC-MS analysis of molecules in observed in aerosols generated from 100 puffs of mango flavor e-liquid, in emissions captured with pads, and two series impingers.

Peak Number	Compound	Retention Index	Retention Time	Library Match	E-Liquid	Impinger	Filter Pad
**1**	Propylene Glycol	724	3.8	97	X	X	X
**2**	1-hexanol	860	4.57	98	X		X
**3**	Methyl hexanoate	884	5.43	95			X
**4**	1,2-propanediol, 2-acetate	864	5.6	87	X	X	
**5**	1,2-propanediol, 2-acetate	864	5.82	96		X	
**6**	Hexyl acetate	984	6.65	96	X		
**7**	Methyl heptanoate	984	6.87	94			X
**8**	Glycerol	967	8.5	98	X	X	X
**9**	Hexyl butanoate	1183	9.21	96	X		
**10**	Citronellol	1179	10.06	96	X		X
**11**	Glyceryl 1-monoacetate	1091	10.45	93		X	
**12**	Hexyl hexanoate	1381	11.83	97	X		X
**13**	Glycerol 1,2-diacetate	1230	11.9	93		X	
**14**	Nicotine	1341	13.04	97	X	X	X
**15**	Piperonal	1326	13.91	97		X	
**16**	γ-Decalactone	1383	15.1	97	X	X	X
**17**	δ-Decalactone	1404	15.98	98	X	X	X
**18**	γ-Undecalactone	1582	16.85	96	X	X	X
**19**	τ-Cadinol	1580	16.93	88			X
**20**	α-Cadinol	1580	17.02	88			X
**21**	1-Naphthalenol	1580	17.17	92			X
**22**	Diethyl phthalate	1639	17.95	97	X	X	X
**23**	Cadalene	1706	18.36	95			X
**24**	Hexadecanal	1800	18.4	93			X
**25**	Methyl hexadecanoate	1878	19.8	96			X
**26**	octadecanal	1999	21.2	95			X
**27**	Methyl stearate	2077	22.4	96		X	X
**28**	Butyl Hexadecanoate	2177	22.99	94	X		X
**29**	Butyl octadecanoate	2375	25.18	93	X		X
**30**	9-octadecanamide	2228	26.55	91			X

**Table 4:** GC-MS analysis of molecules in observed in aerosols generated from 100 puffs of cinnamon flavor e-liquid, in emissions captured with pads, and two series impingers.

Peak Number	Compound	Retention Index	Retention Time	Library Match	E-Liquid	Impinger	Filter Pad
**1**	Propylene Glycol	724	3.8	98	X	X	X
**2**	1-(2-methoxy-1-methylethoxy)propane-2-ol	967	6.96	98			X
**3**	Glycerol	967	8.5	97	X	X	X
**4**	Cinnamaldehyde	1189	12.64	97	X	X	X
**5**	Nicotine	1341	13.03	97	X	X	X
**6**	Ethyl Vanillin	1491	15.6	97	X	X	X
**7**	Diethyl phthalate	1639	17.9	97	X	X	X
**8**	Methyl hexadecanoate	1878	19.8	96			X
**9**	Methyl sterate	2077	22.37	96			X
**10**	Tetracosyl acetate	2773	23.28	95	X		
**11**	Tributyl prop-1-ene-1,2,3-tricarboxylate	2297	24.3	93		X	
**12**	Butyl octadecanoate	2375	25.18	93	X		
**13**	Tributyl acetylcitrate	2594	25.52	92		X	

**Table 5:** Compounds observed in e-liquid, impinger, and filter pads.

Compound	Flavor
Propylene Glycol	All
Glycerol	All
Ethyl maltol	Vanilla
Cinnamaldehyde	Cinnamon
Nicotine	All
γ-Decalactone	Mango
Vanillin	Vanilla
Ethyl Vanillin	Vanilla
δ-Decalactone	Mango
γ-Undecalactone	Mango
Diethyl phthalate	All
Piperonal propylene glycol acetal	Vanilla
Butyl hexadecanoate	Vanilla

**Table 6:** Compounds observed in e-liquid and impinger.

Compound	Flavor
1,2-propanediol, 2-acetate	All
Glyceryl 1-monoacetate	Vanilla, Mango

**Table 7:** Compounds observed in e-liquid and filter pads.

Compound	Flavor
Citronellol	Mango
Hexyl hexanoate	Mango
Butyl hexadecanoate	Mango

**Table 8:** Compounds observed only in the impinger.

Compound	Flavor
Glyceryl 1-monoacetate	Mango
Tributyl prop-1-ene-1,2,3-tricarboxylate	Cinnamon
Tributyl acetylcitrate	Cinnamon

**Table 9:** Compounds observed only in the filter pads.

Compound	Flavor
Methyl hexanoate	Mango
Methyl heptanoate	Mango
1-(2-methoxy-1-methylethoxy)propane-2-ol	Cinnamon
Dodecanol	Vanilla, Mango
Decyl acetate	Vanilla, Mango
τ-Cadinol	Mango
α-Cadinol	Mango
1-Naphthalenol	Mango
Lauryl acetate	Vanilla
Cadalene	Mango
Hexadecanal	Mango
Methyl hexadecanoate	Cinnamon
Octadecanal	Mango
Methyl stearate	Vanilla, Cinnamon
9-octadecanamide	Mango
